# Identification of tumor microenvironment‐based genes associated with acquired resistance to EGFR Tyrosine Kinase Inhibitor in Lung Adenocarcinoma

**DOI:** 10.7150/jca.57008

**Published:** 2022-01-01

**Authors:** Wenjie Chen, Wen Li, Zhenkun Liu, Guangzhi Ma, Yunfu Deng, Lingling Zhu, Qinghua Zhou

**Affiliations:** 1 Lung Cancer Center, West China Hospital, Sichuan University, Chengdu, China.; 2 Department of Thoracic Surgery, West China Hospital, Sichuan University, Chengdu, China.; 3 Tianjin Key Laboratory of Lung Cancer Metastasis and Tumor Microenvironment, Tianjin Lung Cancer Institute, Tianjin Medical University General Hospital, Tianjin, China.; 4 Department of Thoracic Surgery, The Third Affiliated Hospital of Kunming Medical University (Yunnan Cancer Hospital, Yunnan Cancer Center), Kunming, China.

**Keywords:** lung adenocarcinoma, tumor environment, immune cell infiltration, immunotherapy, EGFR-TKI resistance

## Abstract

**Background:** The tumor microenvironment evidently affects treatment response and clinical outcome. This study aims to construct a tumor microenvironment-based crosstalk between immunotherapy and epidermal growth factor receptor tyrosine kinase inhibitor (EGFR-TKI) in lung adenocarcinoma.

**Methods:** We used ESTIMATE algorithm to calculate stromal and immune scores. Differentially expressed genes (DEGs) were extracted based on the comprehensive analysis of immune score groups and EGFR-TKI resistance samples. The independent prognostic value of the five selected genes was assessed by univariate/multivariate Cox regression analysis, survival analysis and the receiver operating characteristic (ROC) curve. Correlation analysis was performed using Spearman's rho value through TIMER 2.0.

**Results:** The Kaplan-Meier survival curve show that patients with higher immune scores have significantly better overall survival. We identified 1328 DEGs from immune score groups and 806 DEGs from the EGFR-TKI resistance cohort GSE123066. A total of 19 co-regulated genes were found, and the Cox regression model produced a significant statistical prognosis for five genes (*CENPF*, *CYSLTR1*, *GLDN*, *PIGR* and *SCGB3A1*). Multivariate Cox regression analysis showed that the selected five gene signatures could be used as independent prognostic indicators. Furthermore, GSEA and correlation analysis demonstrated that *CENPF* was positively correlated to the signalling pathway which related to EGFR-TKI resistance and the well-known bypass gene.

**Conclusion:** Our findings indicate that *CENPF*, *CYSLTR1*, *GLDN*, *PIGR* and *SCGB3A1* are independent prognostic biomarkers associated with acquired EGFR-TKI resistance and tumor immune cell infiltration in lung adenocarcinoma, and *CENPF* may be a potential target that can improve immunotherapy efficacy and overcome the acquired EGFR-TKI resistance.

## Introduction

Lung cancer is one of the most frequently diagnosed malignancies and the leading cause of cancer related deaths 1. It is categorised into adenocarcinoma (LUAD), squamous cell type and large cell type, and LUAD accounts for more than 40% of all lung cancer cases 2. It has been reported that the activating mutations in tyrosine kinase domain of EGFR in LUAD were found 10%-15% in the American and Europe and 40%-50% in Asia 3. Small molecule epidermal growth factor receptor tyrosine kinase inhibitors (EGFR-TKIs) are clinically effective for the first-line treatment of EGFR-mutated NSCLC 3-5. However, the efficacy is transient, and the acquired resistance to EGFR-TKI is inevitable after 9-14 months of treatment 6-9.

The tumor microenvironment (TME) is consist of various cells, including endothelial cells, fibroblasts, immune cells and extra-cellular components that surround the tumor cells 10. The TME can critically influence tumor initiation, progression and metastasis and plays a vital role in therapeutic efficacy 11. Oncogenic alterations can promote an immunosuppressive TME through reduced tumor antigen expression and T-cell infiltration in tumor beds 12. Tumor or stromal cells might result in microenvironment-induced drug resistance through secreting soluble factors, and tumor-associated macrophages have been generally considered the main regulators of therapeutic response in the TME 13.

Immune checkpoint plays a prominent role of immune suppression in tumors and their microenvironment 14. In addition, tumors with low levels of immune infiltration are associated with the low rate of response to programmed death-1 (PD-1) inhibitors 15. PD-1 inhibitors, such as nivolumab and pembrolizumab, are another important treatment for NSCLC. Compared with docetaxel, PD-1 inhibitors could prolong the overall survival of NSCLC patients who had been treated with platinum-based doublet chemotherapy 16, 17. Moreover, a previous study found that PD-1 inhibitors are less effective in treating NSCLC patients with EGFR mutations, and low levels of both programmed death-ligand 1 (PD-L1) and CD8+tumor infiltrating lymphocytes in the TME might be the basis of this adverse clinical response 18. Although immune-checkpoint inhibitors and EGFR-TKIs have shown promising clinical results for LUAD 19-21, the link between the two treatment remains unclear.

ESTIMATE is one of the widely used algorithms for quantifying the stromal and immune components in the TME of malignant tumor tissues 22. It has shown effectiveness in a variety of malignancies, including breast cancer, urothelial cancer, multiple myeloma and neck squamous cell carcinoma 23-29

In this study, we estimated the immune components of the TME and the EGFR-TKI resistance related genes and then identified reliable prognostic biomarkers for LUAD. The newly found genes might render tumor cells more sensitive to EGFR-TKIs and immunotherapy for LUAD patients.

## Material and Methods

### Data collection

The gene expression profiles and phenotype data such as pathological factors, and the survival outcome of the LUAD cohort, were obtained from The Cancer Genome Atlas (TCGA) (https://portal.gdc.cancer.gov/). Criteria for patient selection: Primary Site is bronchus and lung; Project ID is TCGA-LUAD; Workflow Type is HTSeq-Counts; Data Category is Transcriptome Profiling; Data Type is Gene Expression Quantification. The profiles of 526 LUAD and 59 adjacent normal lung tissues were included in the study.

Gene set GSE123066 profiles were obtained from Gene Expression Omnibus (GEO, https://www.ncbi.nlm.nih.gov/geo/). According to the selection criteria, three gefitinib-sensitive samples (GSM3494550, GSM3494551, GSM3494552) and three gefitinib-resistant samples (GSM3494553, GSM3494554, GSM3494555) were included.

Independent validation set GSE26939 profiles were obtained from Gene Expression Omnibus (GEO, https://www.ncbi.nlm.nih.gov/geo/).

### Estimation of stromal and immune cells and identification of deferentially expressed genes

The stromal and immune scores of the TCGA data were calculated by using R package 'ESTIMATE' 22. Survival curves were constructed based on Kaplan-Meier (K-M) by using R package 'survival', the log‐rank test *P* < 0.05 was set as the cut-off. The DEGs were screened by using the R package 'limma' 30, | Log2 (fold change) | ≥ 1.0 and *p*-value < 0.05 were set as the cut-off.

### Function and pathway enrichment analysis of DEGs

The gene ontology (GO) analysis and the Kyoto Encyclopaedia of Genes and Genomes (KEGG) pathway enrichment analysis were performed by the R package 'clusterProfiler'^31^, the false discovery rate (FDR) and *p*-value < 0.05 was set as the cut-off.

### Validation of prognostic value of selected genes for LUAD

Univariate Cox hazards regression analysis was used to analyse the commonly regulated genes and confirm their estimated regression coefficients (β), and *p* < 0.05 was considered to have prognostic value. Kaplan-Meier plotter (www.kmplot.com/lung) 32 were used to verify the prognostic value of the selected genes. Immunohistochemical staining of prognostic genes obtained from The Human Protein Atlas (https://www.proteinatlas.org/) 33.

### Prognostic signature construction and risk score calculation

The risk score formula: risk score = β_ gene 1_ × Expression _gene 1_ +β_gene 2_ × Expression _gene 2_ +... +β_gene [n]_ × Expression _gene [n]_. LUAD patients above the median risk score would be divided into the high-risk group, and the rest would be divided into the low-risk group. The ROC based on three-year survival and K-M survival curves were utilised to evaluate the diagnostic efficacies. The effects of risk score and clinicopathological variables on the overall survival were confirmed by multivariate Cox hazards regression analysis.

### GSEA enrichment analysis

Among the 526 LUAD-TCGA samples, the top 100 samples (*CENPF*_pos) exhibited high levels of *CENPF* expression, and the bottom 100 samples (*CENPF*_neg) displayed low levels of *CENPF* expression. Then, we performed GSEA with the signal-to-noise measure to rank the genes in terms of their association with the LUAD groups (*CENPF*_pos vs. *CENPF*_neg).

### PPI network construction and module analysis

The protein-protein interaction (PPI) network was obtained from the STRING database 34 (http://string-db.org). The PPI network was subsequently visualised using the Cytoscape 35 software, and the “cytohubba” plug-in was used for modular analysis to identify the top 10 hub genes.

### Correlation analysis

The correlation between *CENPF* and the EGFR-TKI resistance associated genes 6 in the LUAD was identified through TMIE2.0 (https://cistrome.shinyapps.io/timer/), with the Spearman's rho value and the estimated statistical significance 36, 37.

## Results

**Figure 1:**
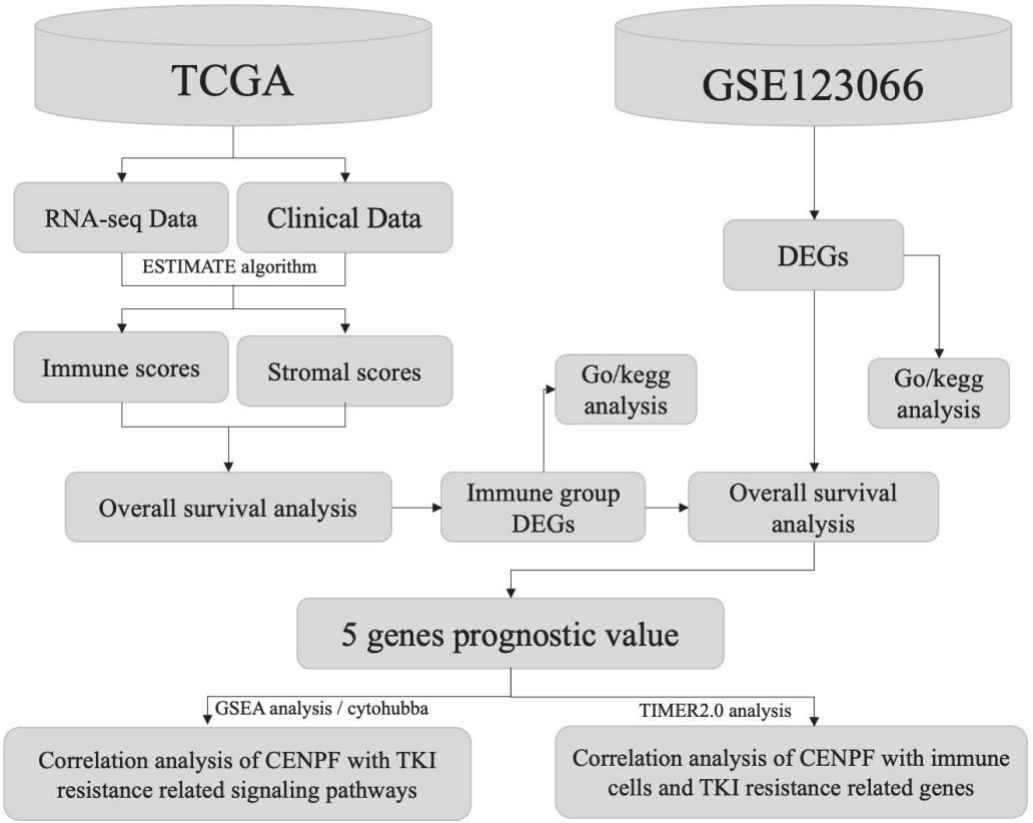
Workflow of the current work.

Workflow of the current work is displayed in Figure 1.

### Tumor progression was associated with immune scores

**Figure 2:**
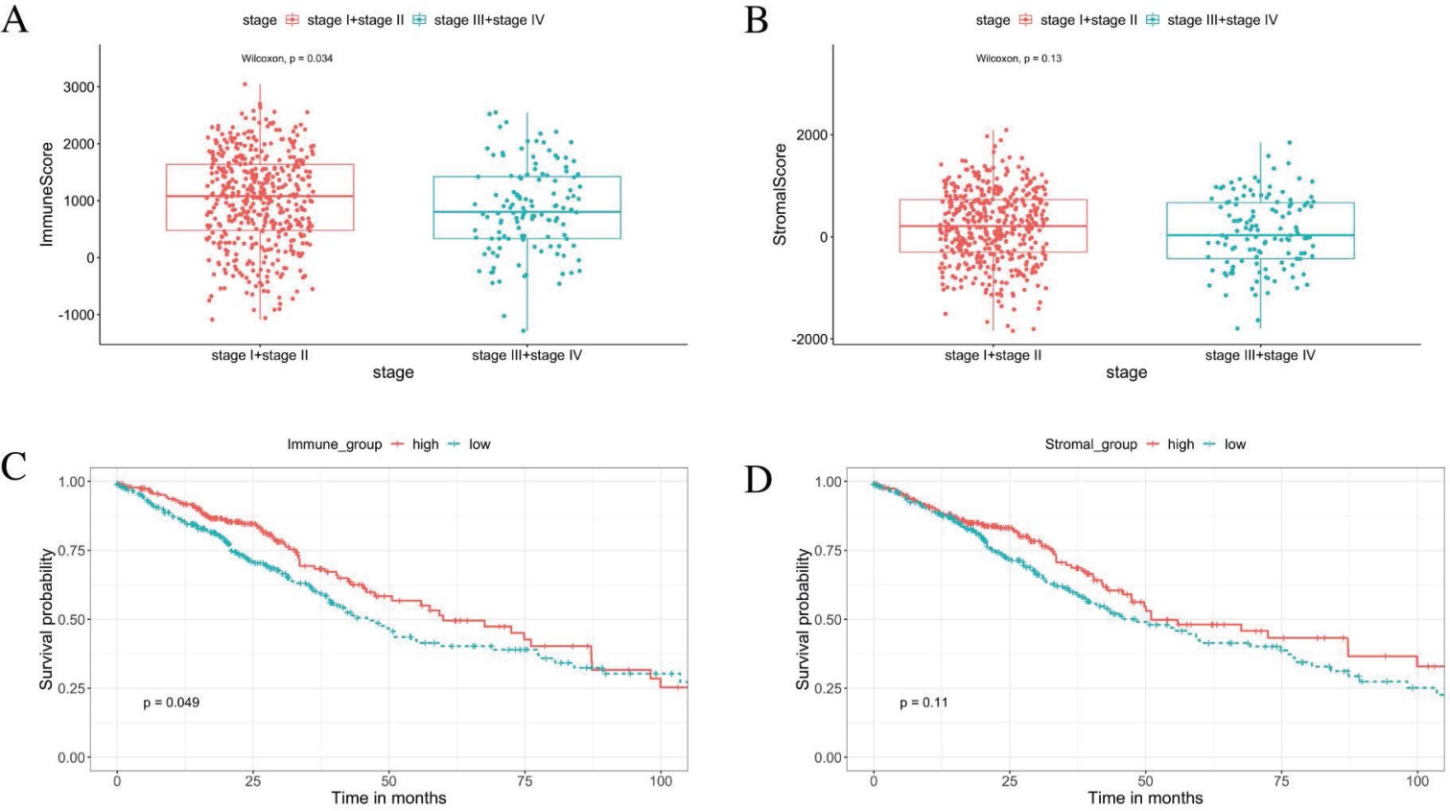
** Immune scores are associated with stage of LUAD progression and their overall survival.** (A) The immune score is significantly negative associated with tumor progression (*p* < 0.05). (B) The stromal score is insignificantly associated with the early stage compared with the advanced stage (*p* = 0.13). (C) K-M curves showed that the overall survival of patients in the high immune score group were significantly better than that in the low immune score group (*p* < 0.05). (D) K-M survival curves show that there is no statistically significant difference in the stromal scores groups (*p* = 0.11).

From the ESTIMATE analysis, immune scores ranging from -1284.72 to 3045.14 were generated, while the stromal scores ranged from -1842.88 to 2093.33 for the 585 LUAD patients enrolled in this study. To detect the correlation between the stromal/immune scores and tumor progression. We divided the tumor stage into stage I + stage II and stage III + stage IV groups. The result shows the tumor progression is significantly negative correlated with the immune scores. The patients in the stage I + stage II group obtained higher immune scores than those in the stage III + stage IV group (Figure 2A, *p* < 0.05). There was no significant association between the stromal scores and tumor progression (Figure 2B, *p* = 0.13).

### Immune score was positively correlated with overall survival

Based on the median value of the stromal and immune scores, we divided the 526 patients into the high score groups and low score groups. The K-M survival curves showed that the overall survival of patients in the high immune score group were significantly better than that in the low immune score group (Figure 2C, log‐rank test *p* < 0.05). Furthermore, there was no statistical difference in overall survival between high and low stromal score groups (Figure 2D, log‐rank test *p* = 0.11). The correlations of the immune score groups and the clinicopathological variables are summarised in Table 1.

### Validation of the LUAD datasets from TCGA and identification of different expression genes based on the immune score groups

**Figure 3:**
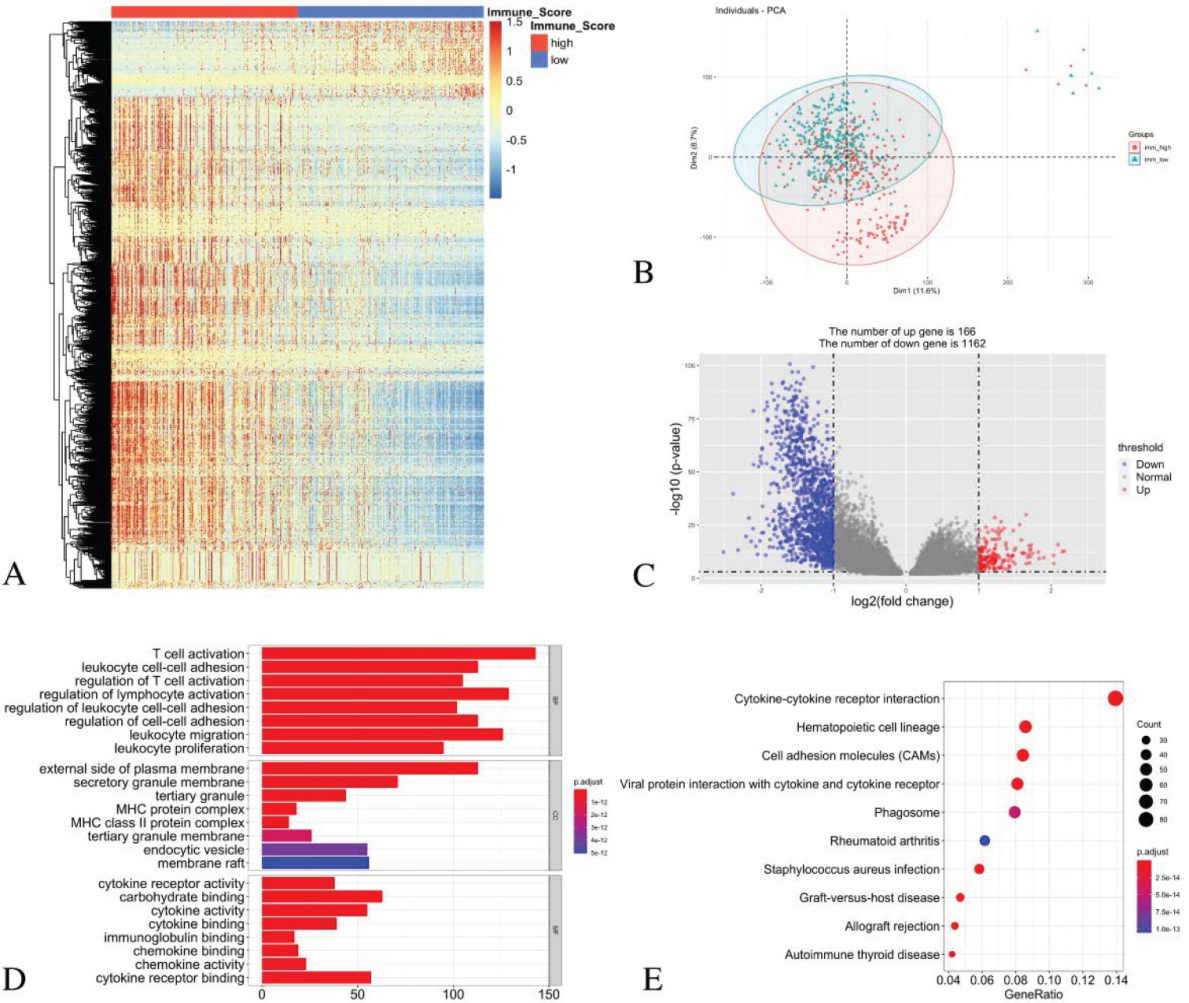
** Identification of DEGs with immune scores in LUAD and GO/ KEGG pathway enrichment analysis.** (A) Heatmap of DEGs with immune scores. (B) PCA of samples with immune scores in LUAD. (C) The volcano graph shows the distribution of DEGs based on the immune score. The X axis represents the fold changes of DEGs, and the Y axis represents the adjusted *p*-value. Red dots present up-regulated genes and blue dots present down-regulated genes (log2 fold change >1.0, *p*-value < 0.05). (D) GO enrichment analysis of DEGs. (E) The KEGG pathway enrichment analysis of DEGs.

The principal component analysis (PCA) result indicates an acceptable intra-group data repeatability for immune scores. The distances between samples in the low immune scores group were short, while those between samples in the high immune scores group were also short in dimension-1 (Dim1) (Figure 3B). From the comparison of the low and high immune score groups, we identified 1328 different expression genes, including 166 up-regulated genes and 1162 down-regulated genes. The heatmap (Figure 3A) and the volcano plot (Figure 3C) show the representatives of the DEGs.

### GO and KEGG enrichment analyses

For the biological processes (BPs), the DEGs were primarily enriched in the T cell activation, the leukocyte cell-cell adhesion, the regulation of T cell activation, the regulation of lymphocyte activation, the regulation of leukocyte cell-cell adhesion, the regulation of cell-cell adhesion, leukocyte migration and leukocyte proliferation. For the cell component (CC), the DEGs were primarily enriched in the external side of the plasma membrane, the secretory granule membrane, the tertiary granule MHC protein complex, the MHC class II protein complex, the tertiary granule membrane, the endocytic vesicle and the membrane raft. For the molecular function (MF), the DEGs were mainly enriched in the cytokine receptor activity, the carbohydrate binding, the cytokine activity, the cytokine binding, the immunoglobulin binding, the chemokine binding, the chemokine activity and the cytokine receptor binding (Figure 3D).

The KEGG pathway analysis showed that all the DEGs were primarily clustered in the cytokine-cytokine receptor interaction, the hematopoietic cell lineage, the cell adhesion molecules, the viral protein interaction with cytokine and cytokine receptor, phagosome, rheumatoid arthritis, staphylococcus aureus infection, graft-versus-host disease, allograft rejection and autoimmune thyroid disease (Figure 3E).

### Identification of DEGs in GSE123066 cohort and function enrichment analysis

**Figure 4:**
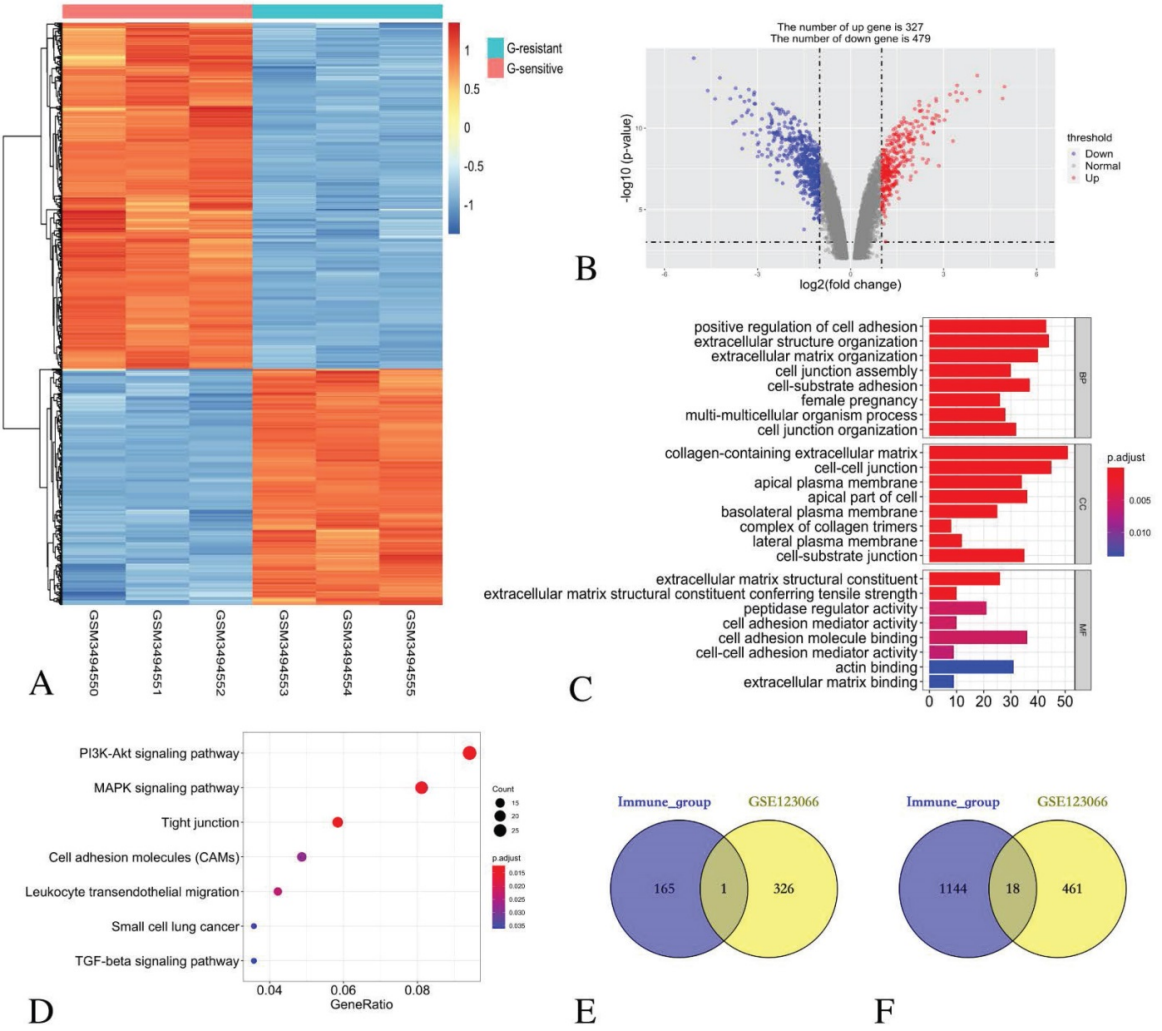
** Identification of DEGs in GSE123066 and GO/ KEGG pathway enrichment analysis of DEGs.** (A) Heatmap of DEGs with immune scores. (B) Volcano maps show the distribution of DEGs. The X axis represents the fold changes of DEGs, and the Y axis represents the adjusted *p*-value. Red dots present up-regulated genes and blue dots present down-regulated genes (log2 fold change >1.0, *p*-value < 0.05). (C) GO enrichment analysis of DEGs. (D) The KEGG pathway enrichment analysis of DEGs. (E) Venn diagram shows the number of commonly up-regulated gene in low immune score group and EGFR-TKI resistance group. (F) Venn diagram shows the number of commonly down-regulated gene in low immune score group and EGFR-TKI resistance group.

By comparing three Gefitinib-resistant samples and three Gefitinib-sensitive samples, 806 DEGs were identified, including 327 up-regulated genes and 479 down-regulated genes. The heatmap (Figure 4A) and the volcano plot (Figure 4B) show the representatives of the DEGs.

GO functional enrichment analysis revealed that the DEGs in the BP category were mainly enriched in the positive regulation of cell adhesion, extracellular structure organisation, extracellular matrix organisation, cell junction assembly, cell-substrate adhesion, female pregnancy, multi-multicellular organism process and cell junction organisation terms. For the enriched CC terms, the DEGs were primarily enriched in the collagen-containing extracellular matrix, the cell-cell junction, the apical plasma membrane, the apical part of the cell, the basolateral plasma membrane, the complex of collagen trimers, the lateral plasma membrane and the cell-substrate junction. For enriched MF terms, the DEGs were primarily enriched in cell adhesion molecule binding, extracellular matrix structural constituent, peptidase regulator activity, extracellular matrix structural constituent conferring tensile strength, cell adhesion mediator activity, cell-cell adhesion mediator activity, actin binding and extracellular matrix binding (Figure 4C).

The KEGG pathway revealed that all the DEGs were primarily enriched in the PI3K-Akt signalling pathway, the MAPK signalling pathway, the tight junction, the cell adhesion molecules, the leukocyte transendothelial migration, small cell lung cancer and the TGF-beta signalling pathway (Figure 4D).

### Identification of common DEGs in immune score group and EGFR-TKI resistance group

The comparison based on immune scores and EGFR-TKI resistance indicates that 166 genes were up-regulated in the low score group, and 327 genes were up-regulated in the Gefitinib resistant group. Venn diagram revealed that only one gene *CENPF* was simultaneously contained within the two examined datasets (Figure 4E).

Similarly, 1162 genes were down-regulated in the low score group, and 479 genes were down-regulated in the Gefitinib resistance group. A Venn diagram reveals that 18 genes, namely, *LCP1, CD14, SPOCK2, LMO2, CYSLTR1, FRMD3, GLDN*, *DHRS9*, *TMEM100, SERPINA1, CST6, PIGR, SCGB3A1, MMP7, SHISA3* and *AZGP1*, were simultaneously contained within the two examined datasets (Figure 4F).

### Filter out prognostic genes from common DEGs and constructed prognostic risk signature model

**Figure 5:**
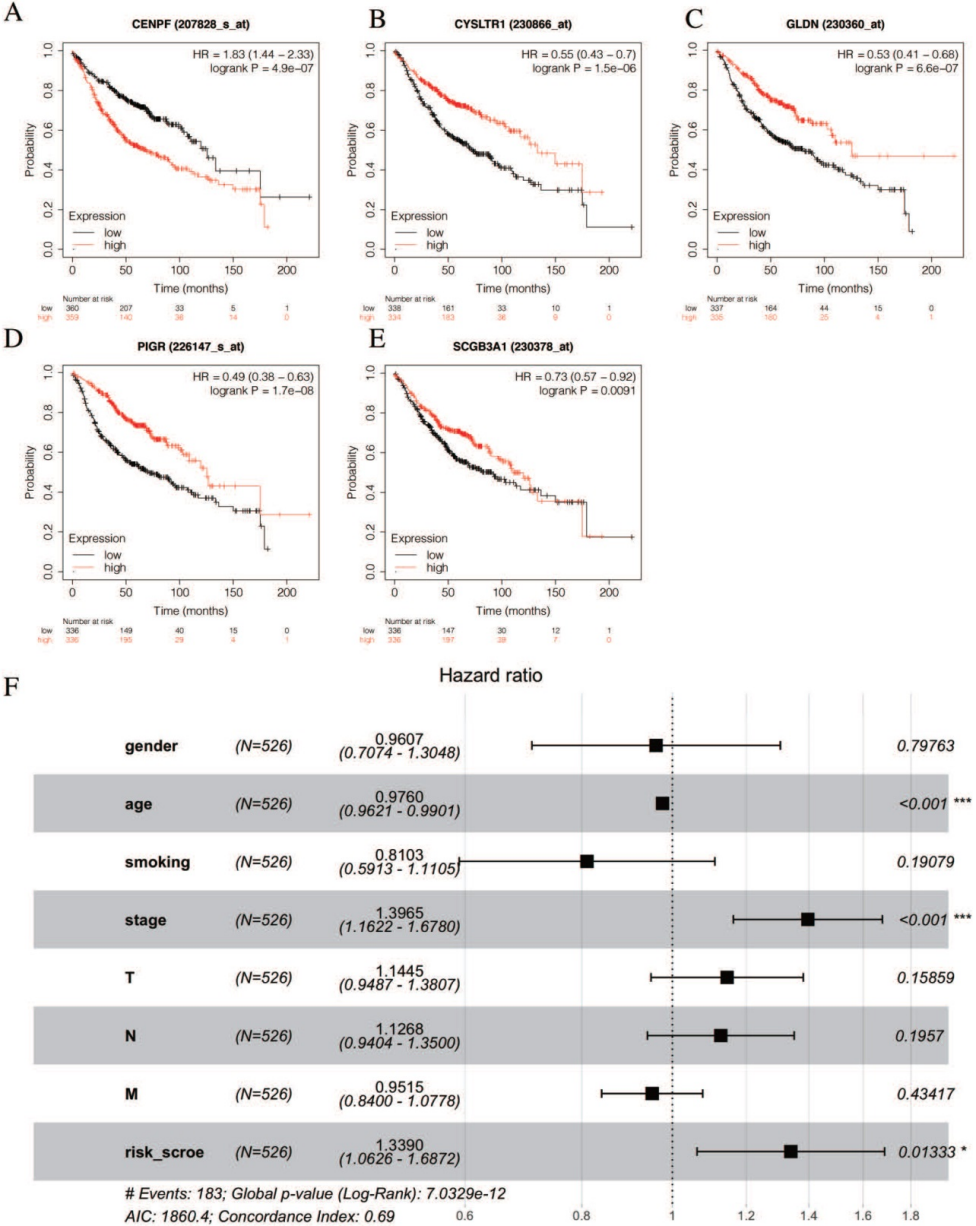
** Validation of selected prognostic genes for LUAD** (A-E) K-M survival curves for each selected DEGs. Red curves represent high level of gene expression and black curves represent low level of gene expression in LUAD (log-rank test *p* < 0.05). Overall survival in months. (F) Multivariate Cox regression analysis of the association between clinicopathological factors and risk score.

The univariate Cox hazards regression analysis revealed that five genes (i.e., *CENPF*, *CYSLTR1*, *GLDN*, *PIGR* and *SCGB3A1)* were significantly related to the OS and their estimated regression coefficients were confirmed (*P* < 0.05, Table 2). Then, we constructed the K-M survival curves to validate the prognostic value of the selected genes. Remarkably, the high levels of *CEBPF* expression could significantly predict a poor OS, and the low levels of the *CYSLTR1*, *GLDN*, *PIGR* and *SCGB3A1* expressions could significantly predict a poor OS (log-rank test *p* < 0.05, Figures 5A-E). Multivariate Cox regression analysis was performed with the following factors: gender, age, smoking history, AJCC stage and risk score. The results show that the risk score is still significantly related to OS (Figure 5F).

**Figure 6:**
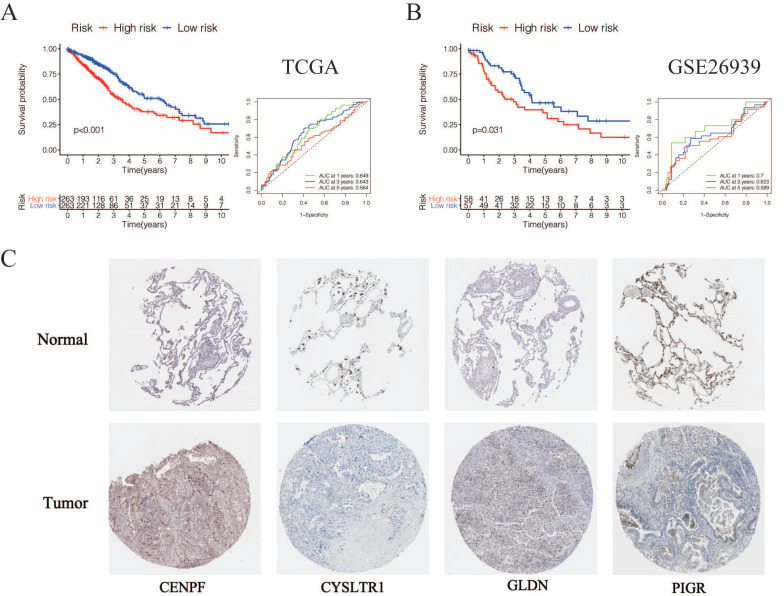
** Validation of selected prognostic genes for LUAD** (A) K-M overall survival curve for risk score groups and ROC curves in the TCGA dataset (*p* < 0.05). (B) K-M overall survival curve for risk score groups and ROC curves in the GSE26939 (*p* < 0.05). (C) Immunohistochemical staining analysis of prognostic genes in lung cancer tissues from The Human Protein Atlas.

According to the following formula: risk score = (0.15 × Exp *_CENPF_*) + (-0.12 × Exp*_CYSLTR1_*_)_ + (-0.09 × Exp*_GLDN_*) + (-0.065 × Exp*_PIGR_*) + (-0.069 × Exp*_SCGB3A1_*), we calculated the risk scores for LUAD patients in the TCGA. The sets were divided into the high- (n = 263) and low-risk groups (n = 263). Based on the median risk score, 263 patients were divided into high-risk group and the rest into low-risk group. The K-M curve shows that the OS of the low-risk group is significantly higher than the high-risk group (*p* < 0.05, Figure 6A). The K-M curve and ROC curves of the independent validation set GSE26939 showed that the OS of the low-risk group is significantly higher than the high-risk group (*p* < 0.05, Figure 6B). Immunohistochemical staining analysis of prognostic genes in lung cancer tissues from The Human Protein Atlas showed that the expression levels of *CENPF* were significantly higher than that in the normal lung tissue, the expression levels of *CYSLTR1* were significantly lower than that in the normal lung tissue, the expression levels of *GLDN* were significantly higher than that in the normal lung tissue and the expression levels of *PIGR* were significantly lower than that in the normal lung tissue (Figure 6C). The expression levels of *SCGB3A1* were not provided in The Human Protein Atlas.

### GSEA analysis of *CENPF* with EGFR-TKI resistance related pathways and genes

We ranked 526 LUAD samples by their relative *CENPF* expression in the TCGA dataset and compared the top 100 samples (*CENPF*_High) and the bottom 100 samples (*CENPF*_Low) through GSEA KEGG enrichment analysis. Given the limited space, only the top 20 pathways are listed in Table 3 (NOM *p*-value < 0.05, FDR < 0.25).

**Figure 7:**
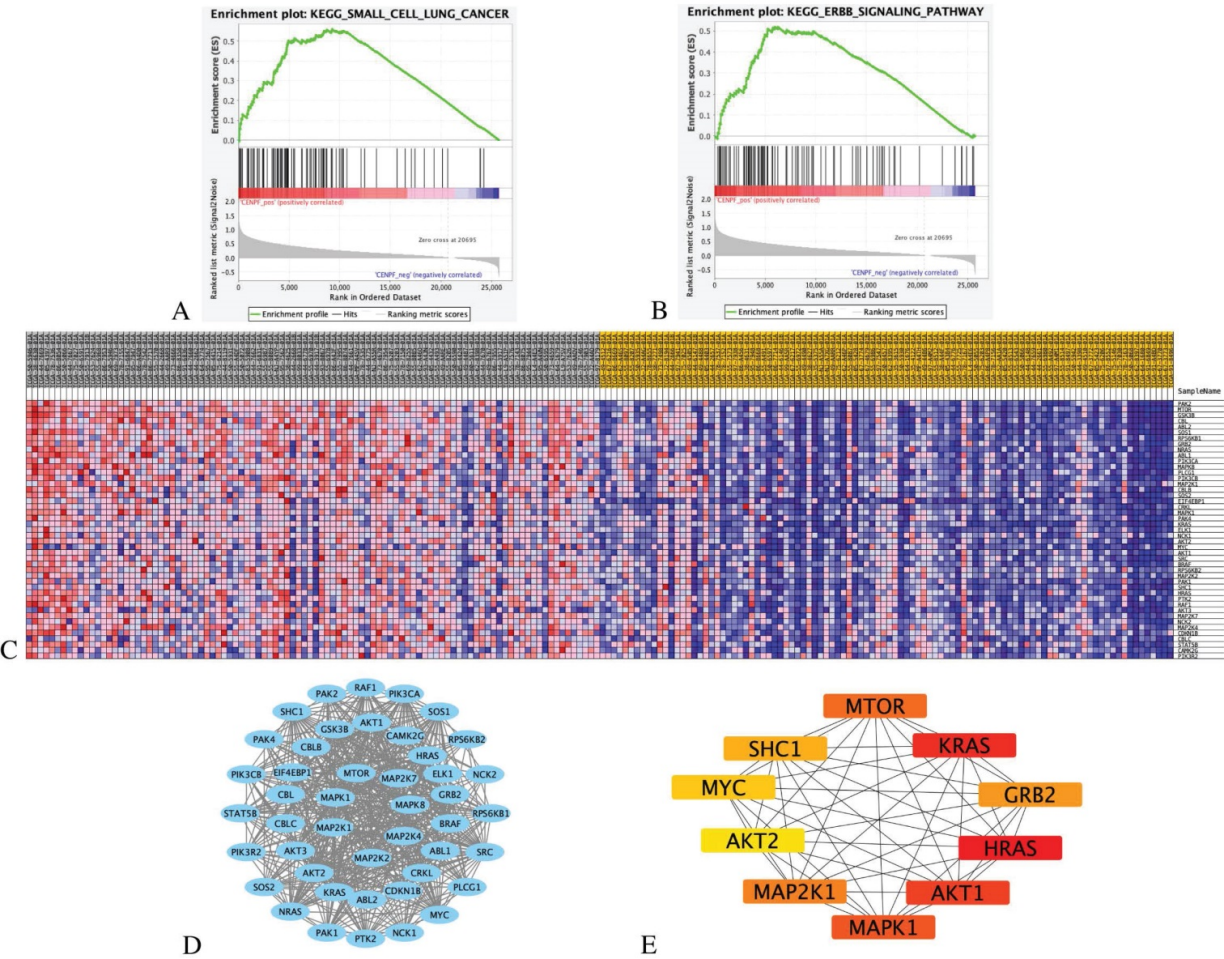
** GSEA analysis of *CENPF* with EGFR-TKI resistance related pathways and genes.** (A) GSEA indicated significant enrichment of EGFR-TKI resistance related pathway, small cell lung cancer pathway in the high expression level of *CENPF*. (B) ErbB signalling pathway in the high expression level of *CENPF*. (C) Heatmap of 45 core genes for enrichment of ErbB signalling pathway. (D) Based on the STRING database, a core genes PPI network was constructed. (E) Top 10 hub genes from core genes ranked by CytoHubba methods.

The results indicate that the *CENPF*_High tumor samples enrich the gene signatures associated with “small cell lung cancer” and “ErbB signalling pathway” compared with *CENPF*_Low samples (Figures 7A-B). The transcriptional expression profiles of the 45 core genes in the ErbB signalling pathway are presented in a heatmap (Figure 7C). To identify the significant module, the STRING online database and Cystoscope software were used to merge the 45 core genes. The PPI network of the core genes was constructed (Figure 7D), and the most significant module was obtained using the Cystoscope plug-in 'cytohubba' (Figure 7E and Table 4).

### Correlation analysis of *CENPF* expression with tumor infiltering immune cells and EGFR-TKI resistance related genes

**Figure 8:**
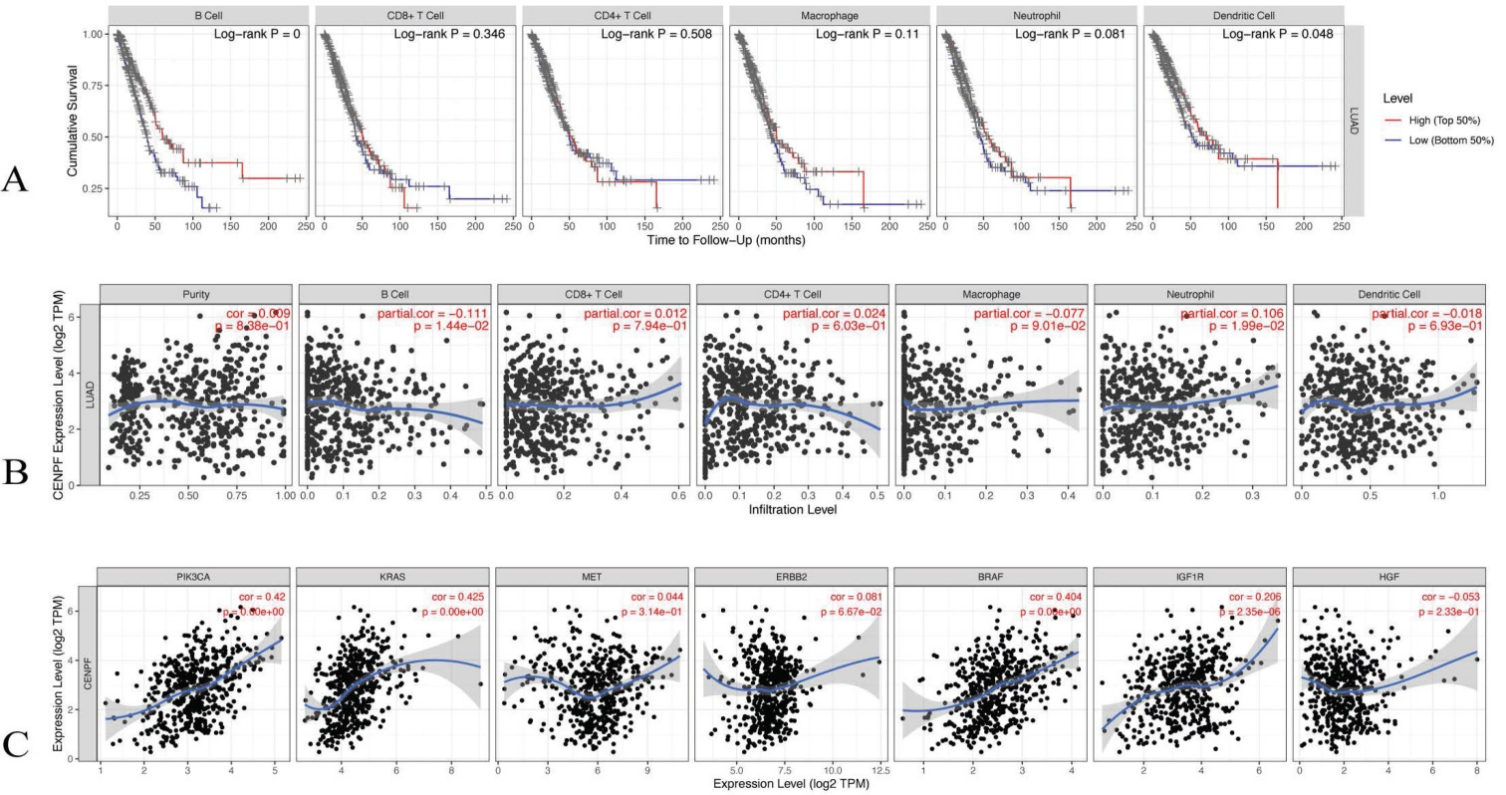
**Correlation analysis of CENPF expression with tumor infiltering immune cells and EGFR-TKI resistance related genes.** (A) Cumulative survival was significantly related to the B cell infiltering level and dendritic cell infiltering level (log-rank test *p* < 0.05). (B) *CENPF* expression was negatively associated with infiltering B cells (Cor = -0.111, *p* < 0.05) and macrophage (Cor = - 0.077, *p* < 0.05). (C) *CENPF* expression was positive correlated with *PIK3CA* (Cor = 0.42, p < 0.05), *KRAS* (Cor = 0.425, *p* < 0.05), *BRAF* (Cor = 0.404, *p* < 0.05) and *IGF1R* (Cor = 0.206, *p* < 0.05).

The TIMER database was utilised to evaluate the correlations of the *CENPF* expression with the tumor infiltering immune cells and the known EGFR-TKI resistance related genes. The infiltering levels of the B cells and the dendritic cell were associated with cumulative survival, that is, a high level predicts good prognosis (log-rank test *p* < 0.05, Figure 8A). The *CENPF* expression was positively associated with infiltering neutrophil (Cor = 0.106, *p* < 0.05), whereas the *CENPF* expression was negatively associated with infiltering B cells (Cor = -0.111, *p* < 0.05) and Macrophage (Cor = -0.077, *p* < 0.05, Figure 8B). Moreover, a positive correlation existed between the *CENPF* expression and the known EGFR-TKI resistance related genes, namely, *PIK3CA* (Cor = 0.42, *p* < 0.05), *KRAS* (Cor = 0.425, *p* < 0.05), *BRAF* (Cor = 0.404, *p* < 0.05) and *IGF1R* (Cor = 0.206, *p* < 0.05, Figure 8C).

## Discussion

In the current work, we tried to identify prognostic genes based on the TME which may be related with the EGFR-TKIs resistance and the efficiency of immunotherapy in LUAD.

First, we find immune score was positively correlated with overall survival and advanced tumor stage have a lower immune score than the early stage. Immune suppression leads to tumor progression via modulating the TME in various ways, such as recruitment of immunosuppressive cells, tumor-associated myeloid-derived suppressor cells, tumor-associated macrophages, and Tregs to tumor sites by migratory and survival factors 38. Then, we identified 1328 DEGs and GO term analysis revealed that many of these DEGs were related to the TME which suggested the immune cells played an important role in LUAD and the extracellular matrix molecules was closely associated with the establishment of the LUAD 39, 40. In addition, KEGG pathway enrichment analysis shows that the DEGs were mainly involved in cell adhesion molecules and cytokine-cytokine receptor interaction, suggesting that the immune system was critical to form the complex LUAD tumor-microenvironment 41, 42. These findings indicate that the up-regulation of the DEG in the low immune score group might be associated with the regulation of the TME, and immunosuppression was formed through this regulation, promoting tumor progression.

Next, we analysed 806 DEGs from EGFR-TKI resistance cohort GSE123066. GO term analysis reveals that many of the DEGs to be enriched in the positive regulation of cell adhesion and extracellular matrix organisation, collagen-containing extracellular matrix and cell-cell junction, and extracellular matrix structural constituent. Consistent with previous studies, the extracellular matrix serves as a microenvironmental clue to promoting EGFR-TKI resistance in lung cancer 43, 44. Moreover, KEGG pathway enrichment reveals that the DEGs were mainly enriched in the PI3K-Akt signalling pathway, the MAPK signalling pathway, the tight junction, the cell adhesion molecules, leukocyte transendothelial migration, small cell lung cancer and the TGF-beta signalling pathway, and those pathways have been shown to be closely associated with promoting the acquired EGFR-TKI resistance and the TME 4, 45, 46.

We then identified 19 common DEGs that were involved in the immune score group and the EGFR-TKI resistance cohort GSE123066. Five of these common DEGs were proved to be related with the overall survival. *CENPF*, *CYSLTR1*, *GLDN*, *PIGR* and *SCGB3A1* were selected as the prognostic biomarkers associated with TME immune cell infiltration and the acquired EGFR-TKI resistance.

We are particularly interested in *CENPF* which is the expression up-regulated in the low immune score group and EGFR-TKI resistance samples, and the expression level of *CENPF* was negatively related with the overall survival for LUAD. Centromere Protein F (*CENPF*) is related to the cell cycle and cell proliferation in several malignant tumors 47. A previous study showed that the high expression level of *CENPF* in NSCLC indicated a poor clinical prognosis 48.

To further identify the functions of *CENPF* in LUAD, we performed KEGG enrichment by GSEA method. The result showed that small cell lung cancer and the ErbB signalling pathway were obviously enriched in *CENPF* high expression phenotype. These results are consistent with the GSE123066 KEGG analysis results, indicating that the high expression level of *CENPF* is related to the promotion of acquired EGFR-TKI resistance. Additionally, 45 core genes were identified in the enrichment of the ErbB signalling pathway, and 10 hub genes (i.e., *HRAS, KRAS, AKT1, MAPK1, MTOR, MAP2K1, GRB2, SHC1, MYC, AKT2*) were screened according to the Cytoscape plug-in cytoHubba. The mutations of *KRAS*, *AKT1* and *MAPK1* have been reported as bypass mutations which are another common mechanism of the acquired EGFR-TKI resistance that can activate the same key downstream effectors as EGFR, thereby promoting the growth and survival of tumor cells 45, 49. Downstream of PI3K-AKT, increased mTOR expression is related to EGFR-TKI resistance in clinical samples 50.

The TIMER database shows that the high level of B cell infiltration is associated with good cumulative survival rate, and there is a significant negative correlation between the *CENPF* expression and the B cell infiltration. These findings indicate that *CENPF* may regulate the immune cell infiltration in LUAD. Furthermore, the *CENPF* expression was positively related with* PIK3CA, KRAS, BRAF* and* IGF1R* which are bypass mutations identified in acquired EGFR-TKI resistance patients, and all of them predicted a poor response to EGFR-TKI therapy 51-54. These results validate the probable function of *CENPF* in promoting acquired EGFR-TKI resistance.

In conclusion, we extracted a series of genes related to the tumor microenvironment based on the immune score calculated by the ESTIMATE algorithm. Then we used GEO dataset to filter out genes related to EGFR-TKI resistance. Survival analysis and Cox hazards regression analysis were performed to validate prognostic value. Finally, we systematically identified that *CENPF*, *CYSLTR1*, *GLDN*, *PIGR* and *SCGB3A1* are novel independent prognostic biomarkers associated with acquired EGFR-TKI resistance and immune infiltration for LUAD patients. Furthermore, GSEA analysis and TIMER2.0 were performed to detect the correlation of *CENPF* with bypass genes and tumor-infiltrating immune cells. However, further mechanism investigation has not been conducted, we believe these genes may provide novel insights for new targets to overcome EGFR-TKI resistance and regulation of the immune infiltration in LUAD.

## Figures and Tables

**Table 1:** Immune score expression and clinicopathological factors in TCGA cases.

Characteristics	Immune score		*P-*value
	High (n=235)	Low (n= 291)	
**Age**			0.0104
≤65	94	154	
>65	130	129	
unknown	11	8	
**Smoking History**			0.0175
smoked	113	171	
non- smoked	122	120	
**Gender**			0.0354
male	97	147	
female	138	144	
**Pathologic Stage**			0.0341
I	141	145	
II	56	66	
III	26	58	
IV	9	17	
no reported	3	5	
**T_stage**			0.0786
T1	85	87	
T2	121	163	
T3	21	27	
T4	5	14	
Tx	3	0	
**N_stage**			0.0751
N0	161	180	
N1	43	52	
N2	22	52	
N3	1	1	
Nx	8	6	
**M_stage**			0.381
M0	158	196	
M1	8	17	
Mx	69	78	

**Table 2:** Identification of prognostic genes.

Name	Descriptions	Immune score grouplow vs. high		EGFR-TKI groupresistant vs. sensitive		Univariate analysis				

		log2FC	P.Value	log2FC	P.Value	HR	95% CI	logrank P	coef	
CENPF	centromere protein F	1.0418	3.11E-15	1.2755	1.07E-06	1.2	1.1 - 1.3	0.0017	0.15	
CYSLTR1	cysteinyl leukotriene receptor 1	-1.1463	7.25E-30	-2.5646	2.88E-09	0.89	0.8 - 0.98	0.022	-0.12	
GLDN	gliomedin	-1.3117	2.36E-22	-1.5769	2.87E-10	0.91	0.84 - 1	0.044	-0.09	
PIGR	polymeric immunoglobulin receptor	-1.4312	2.01E-09	-1.6955	3.75E-10	0.94	0.9 - 0.98	0.004	-0.065	
SCGB3A1	secretoglobin family 3A member 1	-1.6343	4.55E-09	-5.0593	4.98E-15	0.93	0.9 - 0.97	0.00055	-0.069	

log2FC, log2 fold change; P.Value, Nominal P Value; HR, hazard ratio; CI, confidence interval; coef, regression coefficient.

**Table 3:** KEGG pathway enrichment analysis by GSEA.

KEGG pathway name	NES	NOM p-val	FDR
CELL_CYCLE	2.052	0.000	0.029
LYSINE_DEGRADATION	2.043	0.000	0.016
HOMOLOGOUS_RECOMBINATION	2.014	0.000	0.018
DNA_REPLICATION	1.975	0.000	0.023
OOCYTE_MEIOSIS	1.922	0.000	0.037
MISMATCH_REPAIR	1.897	0.000	0.040
NUCLEOTIDE_EXCISION_REPAIR	1.878	0.000	0.044
UBIQUITIN_MEDIATED_PROTEOLYSIS	1.871	0.000	0.046
BASE_EXCISION_REPAIR	1.861	0.000	0.045
SPLICEOSOME	1.855	0.000	0.043
SMALL_CELL_LUNG_CANCER	1.846	0.000	0.044
PROGESTERONE_MEDIATED_OOCYTE_MATURATION	1.842	0.000	0.042
RNA_DEGRADATION	1.816	0.002	0.052
PURINE_METABOLISM	1.786	0.004	0.068
PYRIMIDINE_METABOLISM	1.775	0.006	0.070
PATHWAYS_IN_CANCER	1.768	0.000	0.070
ONE_CARBON_POOL_BY_FOLATE	1.761	0.004	0.070
CHRONIC_MYELOID_LEUKEMIA	1.756	0.000	0.071
ERBB_SIGNALING_PATHWAY	1.754	0.002	0.069
CYSTEINE_AND_METHIONINE_METABOLISM	1.752	0.004	0.066

NES: normalized enrichment score; NOM p-val: Nominal P Value; FDR: The false discovery rate. Gene sets with NOM p-value < 0.05 and FDR q-value < 0.25 are considered as significant.

**Table 4:** Identification of hub genes by cytoHubba.

Gene Name	MCC	DMNC	MNC	Degree	EPC	Ec Centricity	Bottle Neck	Radiality	Betweenness	Closeness	Stress	Clustering Coefficient
HRAS	4.8356E+11	0.8347	39	39	18.13	0.5	2	2.97727	88.73941	41.5	700	0.57085
KRAS	4.8351E+11	0.8675	37	37	18.23	0.5	1	2.93182	59.99892	40.5	584	0.6036
AKT1	4.8247E+11	0.8229	39	39	18.29	0.5	4	2.97727	88.31896	41.5	702	0.56275
MAPK1	4.7951E+11	0.8515	35	35	17.49	0.5	2	2.88636	79.52542	39.5	588	0.60336
MTOR	4.7521E+11	0.9829	29	29	17.29	0.5	6	2.75	16.07856	36.5	216	0.74138
MAP2K1	4.7029E+11	0.9032	30	30	16.69	0.5	3	2.77273	43.3339	37	352	0.67356
GRB2	4.6987E+11	0.8481	37	37	17.89	0.5	4	2.93182	76.8446	40.5	590	0.59009
SHC1	4.4966E+11	0.9771	30	30	17.01	0.5	1	2.77273	17.16792	37	242	0.72874
MYC	4.3484E+11	0.9868	26	26	15.4	0.5	1	2.68182	10.86406	35	148	0.77231
AKT2	4.2943E+11	0.9955	24	24	15.28	0.5	1	2.63636	8.93873	34	116	0.80072

## Abbreviations

BP: biological process
CC: cell component
DEG: Differentially expressed gene
EGFR: epidermal growth factor receptor
FDR: false discovery rate
GEO: Gene Expression Omnibus
GO: gene ontology
KEGG: Kyoto Encyclopedia of Genes and Genomes
LUAD: Lung adenocarcinoma
TCGA: The Cancer Genome Atlas
TKI: tyrosine kinase inhibitor
MF: molecular function
NSCLC: Non-small cell lung cancer
OS: Overall survival
PPI: protein-protein interaction
PD-1: programmed death-1
PD-L1: programmed death-ligand 1
ROC: receiver operating characteristic
